# Di-μ-chlorido-bis­[aqua­chloridodimethyl­tin(IV)]–1,4,7,10,13-penta­oxacyclo­penta­decane (1/1)

**DOI:** 10.1107/S1600536812008781

**Published:** 2012-03-03

**Authors:** Mostafa M. Amini, Seik Weng Ng

**Affiliations:** a Department of Chemistry, General Campus, Shahid Beheshti University, Tehran, Iran; b Department of Chemistry, University of Malaya, 50603 Kuala Lumpur, Malaysia; c Chemistry Department, Faculty of Science, King Abdulaziz University, PO Box 80203 Jeddah, Saudi Arabia

## Abstract

The Sn, Cl and water O atoms of the title compound, [Sn_2_(CH_3_)_4_Cl_4_(H_2_O)_2_]·C_10_H_20_O_5_, lie on a special position of 2 site symmetry. The Sn^IV^ atom shows *cis*-C_2_SnCl_2_O trigonal–bipyramidal coordination [C—Sn—C = 157.0 (1)°]; however, two [Me_2_SnCl_2_(H_2_O)] units are linked by a tin–chlorine bridge [Sn←Cl = 3.247 (1) Å] across a center of inversion, generating a dinuclear species, so that the geometry is better regarded as a *mer*-C_2_SnCl_3_O octa­hedron. The crown ether inter­acts through O—H⋯O hydrogen with the metal atom through the coordinated water mol­ecules in an outer-sphere manner, generating a hydrogen-bonded chain running along [101]. The 15-crown-5 mol­ecule is disordered over the 2/*m* site.

## Related literature

For [Me_2_SnCl_2_(H_2_O)_2_]·15-crown-5, see: Amini *et al.* (1994 ▶); Yap *et al.* (1996 ▶).
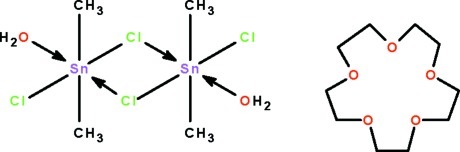


## Experimental

### 

#### Crystal data

[Sn_2_(CH_3_)_4_Cl_4_(H_2_O)_2_]·C_10_H_20_O_5_
*M* *_r_* = 695.61
Monoclinic, 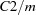

*a* = 14.2351 (13) Å
*b* = 11.4115 (5) Å
*c* = 9.8100 (9) Å
β = 127.183 (14)°
*V* = 1269.6 (3) Å^3^
*Z* = 2
Mo *K*α radiation
μ = 2.42 mm^−1^
*T* = 100 K
0.30 × 0.25 × 0.20 mm

#### Data collection

Agilent SuperNova Dual diffractometer with an Atlas detector
Absorption correction: multi-scan (*CrysAlis PRO*; Agilent, 2011 ▶) *T*_min_ = 0.531, *T*_max_ = 0.644
5824 measured reflections
1524 independent reflections
1495 reflections with *I* > 2σ(*I*)
*R*_int_ = 0.016

#### Refinement

*R*[*F* ^2^ > 2σ(*F* ^2^)] = 0.017
*wR*(*F* ^2^) = 0.042
*S* = 0.99
1524 reflections
93 parameters
43 restraints
H-atom parameters constrained
Δρ_max_ = 0.48 e Å^−3^
Δρ_min_ = −0.72 e Å^−3^

### 

Data collection: *CrysAlis PRO* (Agilent, 2011 ▶); cell refinement: *CrysAlis PRO*; data reduction: *CrysAlis PRO*; program(s) used to solve structure: *SHELXS97* (Sheldrick, 2008 ▶); program(s) used to refine structure: *SHELXL97* (Sheldrick, 2008 ▶); molecular graphics: *X-SEED* (Barbour, 2001 ▶); software used to prepare material for publication: *publCIF* (Westrip, 2010 ▶).

## Supplementary Material

Crystal structure: contains datablock(s) global, I. DOI: 10.1107/S1600536812008781/xu5444sup1.cif

Structure factors: contains datablock(s) I. DOI: 10.1107/S1600536812008781/xu5444Isup2.hkl

Additional supplementary materials:  crystallographic information; 3D view; checkCIF report

## Figures and Tables

**Table 1:** Hydrogen-bond geometry (Å, °)

*D*—H⋯*A*	*D*—H	H⋯*A*	*D*⋯*A*	*D*—H⋯*A*
O1w—H1⋯O1	0.84	2.37	2.753 (4)	108
O1w—H1⋯O1^i^	0.84	2.38	2.753 (4)	107
O1w—H1⋯O2^ii^	0.84	2.12	2.687 (9)	125
O1w—H1⋯O5^iii^	0.84	2.26	2.810 (9)	123

Symmetry codes: (i) 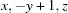; (ii) 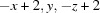; (iii) 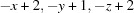.

**Fig. 1.**
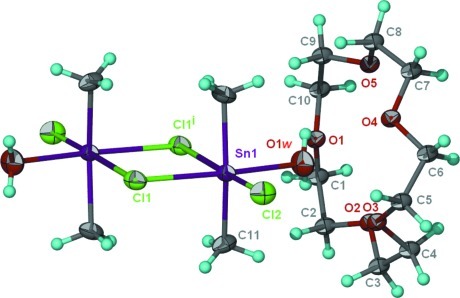
Thermal ellipsoid plot (Barbour, 2001) of [Me2SnCl2(H2O)2]2.15-crown-5 at the 70% probability level; hydrogen atoms are drawn as spheres of arbitrary radius.

**Fig. 2.**
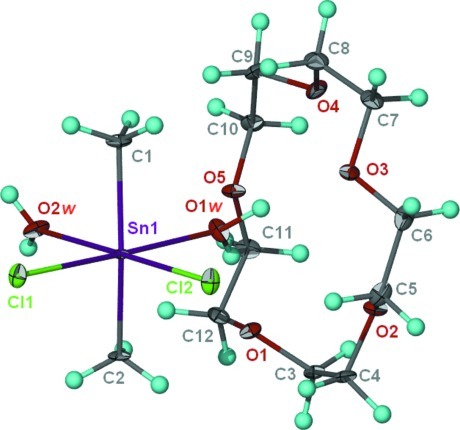
Thermal ellipsoid plot (Barbour, 2001) of [Me2SnCl2(H2O)2].15-crown-5 at the 70% probability level; hydrogen atoms are drawn as spheres of arbitrary radius (Amini & Ng, Unpublished results).

## supplementary crystallographic information

### Comment

Dimethyltin dichloride in the form of its dihydrate forms a 1:1 co-crystal with 15-crown-5; the adduct belongs to the *C*2/*c* space group at room temperature [*a* 9.313 (2), *b* 17.266 (3), *c* 13.525 (3) Å; β 107.37 (2) %]. The Sn^IV^ atom lies on a twofold rotation axis, and the O atoms of the crown ether all point away from the middle of the ring so that all O_water_···O_crown ether_ interactions exceed 3.5 Å (Amini *et al.*, 1994). A later, low-temperature (233 K) study corrected the space group of the room-temperature study to *P*2_1_/*n* because dynamic disorder gave rise to ambiguities in identifying atoms (Yap *et al.*, 1996). In fact, the water molecule does interact with the crown ether. We repeated the synthesis and used chloform as solvent for crystallization in a 100 K study to confirm the hydrogen bonding interactions (Amini & Ng, Unpublished results).

We then used the chloroform-crystallized compound, [Me_2_SnCl_2_(H_2_O)_2_]^.^15-crown-5, in a further recrystalliation from methanol, and we obtained the monoaqua 2:1 adduct (Scheme I). The isolation of the 2:1 adduct is reproducible as a second recrystallization from solvent gave an identical compound, so that attempt represents an example of the influence of solvent in the formation of co-crystals.

In [Me_2_SnCl_2_(H_2_O)]_2_^.^15-crown-5, the Sn^IV^ atom shows *cis*-C_2_SnCl_2_O trigonal bipyramidal coordination [C–Sn–C 157.0 (1) °]; however, two [Me_2_SnCl_2_(H_2_O)] units are linked by a tin–chlorine bridge [Sn←Cl 3.247 (1) Å] across a center-of-inversion to generate a dinuclear species, so that the geometry is better regarded as a *mer*-C_2_SnCl_3_O octahedron (Fig. 1). The crown ether interacts indirectly with the metal atom through the coordinated water molecules in an outer-sphere manner to generate a hydrogen-bonded chain running along [1 0 1] (Table 1).

### Experimental

Dimethyltin dichloride (0.22 g, 1 mmol) and 15-crown-5 (0.24 g, 1 mol) were dissolved in chloroform (20 ml) to give clear solution. Colorless crystals of Me_2_SnCl_2_(H_2_O)_2_^.^15-crown-5 were formed within a day (Amini *et al.*, 1994); the identity was confirmed by a low-temperature diffraction study.

The 1:1 adduct was recrystallized from methanol to yield the [Me_2_SnCl_2_(H_2_O)]_2_^.^15-crown-5 adduct.

### Refinement

Carbon-bound H-atoms were placed in calculated positions [C–H 0.95 to 0.99 Å, *U*_iso_(H) 1.2 to 1.5*U*_eq_(C)] and were included in the refinement in the riding model approximation.

The water H-atom, whose O atom lies on a twofold rotation axis, was similar treated [O–H 0.84 Å] and its temperature factor s were tied by a factor of 1.5 times.

The 15-crown-5 molecule is disordered over the 2/*m* site. The ring was refined as a 15-atom species subject to 1,2 related distances being restrained to 1.50±0.01 Å. The temperature factors of the five O atoms were made identical, as were those of the ten C atoms. The anisotropic temperature factors of the sole C and O atoms were restrained to be nearly isotropic.

The crystal when measured with Cu radiation in place of Mo radiation in the expectation of resolving the disorder gave a marginally worse outcome, probably because of absorption difficulties.

### Crystal data

**Table:**

[Sn_2_(CH_3_)_4_Cl_4_(H_2_O)_2_]·C_10_H_20_O_5_	*F*(000) = 688
*M**_r_* = 695.61	*D*_x_ = 1.820 Mg m^−^^3^
Monoclinic, *C*2/*m*	Mo *K*α radiation, λ = 0.71073 Å
Hall symbol: -C 2y	Cell parameters from 5314 reflections
*a* = 14.2351 (13) Å	θ = 2.5–27.5°
*b* = 11.4115 (5) Å	µ = 2.42 mm^−^^1^
*c* = 9.8100 (9) Å	*T* = 100 K
β = 127.183 (14)°	Block, colorless
*V* = 1269.6 (3) Å^3^	0.30 × 0.25 × 0.20 mm
*Z* = 2	

### Data collection

**Table:**

Agilent SuperNova Dual diffractometer with an Atlas detector	1524 independent reflections
Radiation source: SuperNova (Mo) X-ray Source	1495 reflections with *I* > 2σ(*I*)
Mirror monochromator	*R*_int_ = 0.016
Detector resolution: 10.4041 pixels mm^-1^	θ_max_ = 27.6°, θ_min_ = 2.5°
ω scan	*h* = −16→18
Absorption correction: multi-scan (*CrysAlis PRO*; Agilent, 2011)	*k* = −13→14
*T*_min_ = 0.531, *T*_max_ = 0.644	*l* = −12→12
5824 measured reflections	

### Refinement

**Table:**

Refinement on *F*^2^	Primary atom site location: structure-invariant direct methods
Least-squares matrix: full	Secondary atom site location: difference Fourier map
*R*[*F*^2^ > 2σ(*F*^2^)] = 0.017	Hydrogen site location: inferred from neighbouring sites
*wR*(*F*^2^) = 0.042	H-atom parameters constrained
*S* = 0.99	*w* = 1/[σ^2^(*F*_o_^2^) + (0.023*P*)^2^ + 2.1038*P*] where *P* = (*F*_o_^2^ + 2*F*_c_^2^)/3
1524 reflections	(Δ/σ)_max_ = 0.001
93 parameters	Δρ_max_ = 0.48 e Å^−^^3^
43 restraints	Δρ_min_ = −0.72 e Å^−^^3^

### Fractional atomic coordinates and isotropic or equivalent isotropic displacement parameters (Å^2^)

**Table:**

	*x*	*y*	*z*	*U*_iso_*/*U*_eq_	Occ. (<1)
Sn1	0.655960 (17)	0.5000	0.51206 (2)	0.01800 (8)	
Cl1	0.43822 (7)	0.5000	0.25799 (9)	0.02629 (17)	
Cl2	0.73232 (8)	0.5000	0.35183 (10)	0.02918 (17)	
O1W	0.8420 (2)	0.5000	0.7676 (3)	0.0329 (5)	
H1	0.8512	0.5605	0.8232	0.049*	
O1	0.8868 (4)	0.5018 (14)	1.0827 (5)	0.0187 (5)	0.25
O2	1.0086 (8)	0.6816 (7)	1.0770 (10)	0.0187 (5)	0.25
O3	1.0110 (5)	0.6179 (5)	0.7954 (7)	0.0187 (5)	0.25
O4	1.0459 (5)	0.3741 (5)	0.8555 (7)	0.0187 (5)	0.25
O5	1.0106 (8)	0.3021 (7)	1.1028 (10)	0.0187 (5)	0.25
C1	0.8963 (15)	0.6095 (12)	1.164 (2)	0.0199 (5)	0.25
H1A	0.9664	0.6078	1.2858	0.024*	0.25
H1B	0.8255	0.6215	1.1595	0.024*	0.25
C2	0.9075 (17)	0.7073 (16)	1.072 (3)	0.0199 (5)	0.25
H2A	0.8355	0.7121	0.9520	0.024*	0.25
H2B	0.9183	0.7831	1.1288	0.024*	0.25
C3	1.0255 (10)	0.7633 (8)	0.9801 (15)	0.0199 (5)	0.25
H3A	1.0707	0.8324	1.0511	0.024*	0.25
H3B	0.9482	0.7904	0.8774	0.024*	0.25
C4	1.0921 (13)	0.7001 (12)	0.928 (2)	0.0199 (5)	0.25
H4A	1.1211	0.7565	0.8846	0.024*	0.25
H4B	1.1606	0.6582	1.0273	0.024*	0.25
C5	1.0639 (7)	0.5468 (6)	0.7385 (10)	0.0199 (5)	0.25
H5A	1.1192	0.5954	0.7333	0.024*	0.25
H5B	1.0017	0.5182	0.6215	0.024*	0.25
C6	1.1287 (7)	0.4445 (6)	0.8525 (11)	0.0199 (5)	0.25
H6A	1.1640	0.3978	0.8088	0.024*	0.25
H6B	1.1929	0.4717	0.9695	0.024*	0.25
C7	1.0979 (8)	0.2730 (8)	0.9620 (13)	0.0199 (5)	0.25
H7A	1.1728	0.2947	1.0729	0.024*	0.25
H7B	1.1153	0.2138	0.9063	0.024*	0.25
C8	1.0136 (11)	0.2234 (7)	0.9908 (17)	0.0199 (5)	0.25
H8A	0.9339	0.2159	0.8806	0.024*	0.25
H8B	1.0402	0.1447	1.0436	0.024*	0.25
C9	0.9022 (14)	0.2965 (13)	1.084 (3)	0.0199 (5)	0.25
H9A	0.8978	0.2221	1.1316	0.024*	0.25
H9B	0.8338	0.3004	0.9611	0.024*	0.25
C10	0.9009 (15)	0.3995 (12)	1.179 (2)	0.0199 (5)	0.25
H10A	0.8346	0.3926	1.1861	0.024*	0.25
H10B	0.9756	0.4036	1.2963	0.024*	0.25
C11	0.6536 (2)	0.68104 (19)	0.5528 (3)	0.0271 (5)	
H11	0.6202	0.6940	0.6144	0.041*	
H12	0.6053	0.7215	0.4423	0.041*	
H13	0.7343	0.7118	0.6206	0.041*	

### Atomic displacement parameters (Å^2^)

**Table:**

	*U*^11^	*U*^22^	*U*^33^	*U*^12^	*U*^13^	*U*^23^
Sn1	0.02135 (12)	0.01588 (12)	0.01478 (11)	0.000	0.00988 (9)	0.000
Cl1	0.0286 (4)	0.0210 (3)	0.0177 (3)	0.000	0.0079 (3)	0.000
Cl2	0.0375 (4)	0.0312 (4)	0.0283 (4)	0.000	0.0249 (4)	0.000
O1W	0.0301 (12)	0.0391 (14)	0.0182 (11)	0.000	0.0088 (10)	0.000
O1	0.0216 (11)	0.0195 (12)	0.0150 (15)	−0.0018 (15)	0.0111 (11)	0.0016 (13)
O2	0.0216 (11)	0.0195 (12)	0.0150 (15)	−0.0018 (15)	0.0111 (11)	0.0016 (13)
O3	0.0216 (11)	0.0195 (12)	0.0150 (15)	−0.0018 (15)	0.0111 (11)	0.0016 (13)
O4	0.0216 (11)	0.0195 (12)	0.0150 (15)	−0.0018 (15)	0.0111 (11)	0.0016 (13)
O5	0.0216 (11)	0.0195 (12)	0.0150 (15)	−0.0018 (15)	0.0111 (11)	0.0016 (13)
C1	0.0250 (10)	0.0192 (13)	0.0199 (13)	−0.0014 (16)	0.0159 (9)	0.0018 (15)
C2	0.0250 (10)	0.0192 (13)	0.0199 (13)	−0.0014 (16)	0.0159 (9)	0.0018 (15)
C3	0.0250 (10)	0.0192 (13)	0.0199 (13)	−0.0014 (16)	0.0159 (9)	0.0018 (15)
C4	0.0250 (10)	0.0192 (13)	0.0199 (13)	−0.0014 (16)	0.0159 (9)	0.0018 (15)
C5	0.0250 (10)	0.0192 (13)	0.0199 (13)	−0.0014 (16)	0.0159 (9)	0.0018 (15)
C6	0.0250 (10)	0.0192 (13)	0.0199 (13)	−0.0014 (16)	0.0159 (9)	0.0018 (15)
C7	0.0250 (10)	0.0192 (13)	0.0199 (13)	−0.0014 (16)	0.0159 (9)	0.0018 (15)
C8	0.0250 (10)	0.0192 (13)	0.0199 (13)	−0.0014 (16)	0.0159 (9)	0.0018 (15)
C9	0.0250 (10)	0.0192 (13)	0.0199 (13)	−0.0014 (16)	0.0159 (9)	0.0018 (15)
C10	0.0250 (10)	0.0192 (13)	0.0199 (13)	−0.0014 (16)	0.0159 (9)	0.0018 (15)
C11	0.0345 (12)	0.0181 (10)	0.0345 (12)	−0.0066 (9)	0.0239 (11)	−0.0058 (9)

### Geometric parameters (Å, º)

**Table:**

Sn1—C11	2.108 (2)	C3—H3A	0.9900
Sn1—C11^i^	2.108 (2)	C3—H3B	0.9900
Sn1—O1W	2.296 (2)	C4—H4A	0.9900
Sn1—Cl2	2.3912 (8)	C4—H4B	0.9900
Sn1—Cl1	2.5459 (10)	C5—C6	1.489 (8)
Sn1—Cl1^ii^	3.2467 (9)	C5—H5A	0.9900
O1W—H1	0.8400	C5—H5B	0.9900
O1—C1	1.427 (9)	C6—H6A	0.9900
O1—C10	1.438 (9)	C6—H6B	0.9900
O2—C2	1.440 (10)	C7—C8	1.501 (8)
O2—C3	1.452 (8)	C7—H7A	0.9900
O3—C5	1.430 (7)	C7—H7B	0.9900
O3—C4	1.447 (10)	C8—H8A	0.9900
O4—C7	1.427 (8)	C8—H8B	0.9900
O4—C6	1.442 (7)	C9—C10	1.508 (9)
O5—C8	1.439 (8)	C9—H9A	0.9900
O5—C9	1.439 (9)	C9—H9B	0.9900
C1—C2	1.505 (9)	C10—H10A	0.9900
C1—H1A	0.9900	C10—H10B	0.9900
C1—H1B	0.9900	C11—H11	0.9800
C2—H2A	0.9900	C11—H12	0.9800
C2—H2B	0.9900	C11—H13	0.9800
C3—C4	1.504 (9)		
			
C11—Sn1—C11^i^	157.00 (13)	H4A—C4—H4B	108.6
C11—Sn1—O1W	86.16 (7)	O3—C5—C6	112.7 (5)
C11^i^—Sn1—O1W	86.16 (7)	O3—C5—H5A	109.1
C11—Sn1—Cl2	100.96 (6)	C6—C5—H5A	109.1
C11^i^—Sn1—Cl2	100.96 (6)	O3—C5—H5B	109.1
O1W—Sn1—Cl2	92.01 (7)	C6—C5—H5B	109.1
C11—Sn1—Cl1	92.07 (7)	H5A—C5—H5B	107.8
C11^i^—Sn1—Cl1	92.07 (7)	O4—C6—C5	108.0 (6)
O1W—Sn1—Cl1	170.83 (7)	O4—C6—H6A	110.1
Cl2—Sn1—Cl1	97.16 (3)	C5—C6—H6A	110.1
C11—Sn1—Cl1^ii^	78.93 (6)	O4—C6—H6B	110.1
C11^i^—Sn1—Cl1^ii^	78.93 (6)	C5—C6—H6B	110.1
O1W—Sn1—Cl1^ii^	85.96 (7)	H6A—C6—H6B	108.4
Cl2—Sn1—Cl1^ii^	177.97 (3)	O4—C7—C8	108.9 (7)
Cl1—Sn1—Cl1^ii^	84.87 (3)	O4—C7—H7A	109.9
Sn1—O1W—H1	109.5	C8—C7—H7A	109.9
C1—O1—C10	113.8 (4)	O4—C7—H7B	109.9
C2—O2—C3	113.8 (7)	C8—C7—H7B	109.9
C5—O3—C4	113.4 (6)	H7A—C7—H7B	108.3
C7—O4—C6	113.4 (6)	O5—C8—C7	107.7 (7)
C8—O5—C9	113.4 (8)	O5—C8—H8A	110.2
O1—C1—C2	108.1 (8)	C7—C8—H8A	110.2
O1—C1—H1A	110.1	O5—C8—H8B	110.2
C2—C1—H1A	110.1	C7—C8—H8B	110.2
O1—C1—H1B	110.1	H8A—C8—H8B	108.5
C2—C1—H1B	110.1	O5—C9—C10	107.4 (8)
H1A—C1—H1B	108.4	O5—C9—H9A	110.2
O2—C2—C1	107.1 (8)	C10—C9—H9A	110.2
O2—C2—H2A	110.3	O5—C9—H9B	110.2
C1—C2—H2A	110.3	C10—C9—H9B	110.2
O2—C2—H2B	110.3	H9A—C9—H9B	108.5
C1—C2—H2B	110.3	O1—C10—C9	106.0 (8)
H2A—C2—H2B	108.5	O1—C10—H10A	110.5
O2—C3—C4	107.6 (8)	C9—C10—H10A	110.5
O2—C3—H3A	110.2	O1—C10—H10B	110.5
C4—C3—H3A	110.2	C9—C10—H10B	110.5
O2—C3—H3B	110.2	H10A—C10—H10B	108.7
C4—C3—H3B	110.2	Sn1—C11—H11	109.5
H3A—C3—H3B	108.5	Sn1—C11—H12	109.5
O3—C4—C3	107.0 (9)	H11—C11—H12	109.5
O3—C4—H4A	110.3	Sn1—C11—H13	109.5
C3—C4—H4A	110.3	H11—C11—H13	109.5
O3—C4—H4B	110.3	H12—C11—H13	109.5
C3—C4—H4B	110.3		

Symmetry codes: (i) *x*, −*y*+1, *z*; (ii) −*x*+1, −*y*+1, −*z*+1.

### Hydrogen-bond geometry (Å, º)

**Table:**

*D*—H···*A*	*D*—H	H···*A*	*D*···*A*	*D*—H···*A*
O1w—H1···O1	0.84	2.37	2.753 (4)	108
O1w—H1···O1^i^	0.84	2.38	2.753 (4)	107
O1w—H1···O2^iii^	0.84	2.12	2.687 (9)	125
O1w—H1···O5^iv^	0.84	2.26	2.810 (9)	123

Symmetry codes: (i) *x*, −*y*+1, *z*; (iii) −*x*+2, *y*, −*z*+2; (iv) −*x*+2, −*y*+1, −*z*+2.
